# Supersaturation-controlled morphology and phase evolution of NiCo LDH in controllable and continuous flow synthesis

**DOI:** 10.1371/journal.pone.0335668

**Published:** 2025-10-29

**Authors:** Yan Hu

**Affiliations:** Ningbo Innovation Center, Zhejiang University, Ningbo, China; Birla Institute of Technology and Science Pilani - K K Birla Goa Campus, INDIA

## Abstract

This study demonstrates a controllable synthesis of NiCo layered double hydroxide (LDH) nanoplates via homogeneous coprecipitation in a continuous flow reactor (CFR). The CFR system enables precise control over LDH morphology and size by maintaining constant supersaturation under pseudo-steady-state conditions. We systematically investigated the relationships between supersaturation, nucleation processes, LDH growth, and morphological evolution, identifying distinct thresholds for homogeneous and heterogeneous nucleation. The competition between nucleation and crystal growth drives morphological transitions from isolated nanoplates to nanoflowers with increasing supersaturation. Furthermore, we characterized the phase transition mechanism between coexisting brucite-like and LDH phases. Four key factors governing the transformation of NiCo mixed hydroxides to NiCo LDH were identified: supersaturation level, metal/alkaline ratio, heterogeneous nucleation, and dissolved oxygen concentration. These findings provide fundamental insights into the controlled synthesis of LDH materials with tunable morphologies for potential applications in energy storage and catalysis.

## 1. Introduction

As a large family of two-dimensional materials, layered double hydroxides (LDHs) possess a composition that can be represented by the general formula [M(II)_1-x_M(III)_x_ (OH)₂]^x +^·[A_x/n_]^n-^ [1–3]. The LDH structure is primarily built on stacked brucite-like layers, in which divalent metal cations are octahedrally coordinated by hydroxyl groups; due to the partial substitution of divalent cations by trivalent metal cations, the layers are positively charged, with the charge amount equal to the molar ratio of M³⁺/(M³⁺ + M²⁺). The compensating anions, organic or inorganic, are present in the interlayer region along with water molecules; hydrogen bonding may form between the hydroxyl groups and the interlayer components, resulting in weak interlayer interactions and thereby feasible expansion properties.

Owing to the tunability and versatility in the types of metal cations (M = Fe, Mg, Ni, Co, Al, etc.), the M³⁺/M²⁺ molar ratio (generally x = 0.2–0.33), and the interlayer galleries, a variety of host-guest assemblies can be established, increasing the compositional and structural complexity of LDHs and thus endowing them with abundant physical and chemical properties. Therefore, LDHs have been extensively utilized as active materials, precursors, and composites, paving their way for a wide range of applications, e.g., in electrochemistry [4–6], photochemistry [7], and pharmaceuticals [8–11].

The typical fabrication methods of LDHs are coprecipitation [12–15] and homogeneous precipitation [16,17] via urea or hexamethylenetetramine hydrolysis. The most common procedure for the former is to mix the cations with a base in the presence of desired intercalated anions at a constant pH, with the advantage of minimizing unwanted metal hydroxides; the latter benefits from the controllable and uniform release of hydroxyl groups through temperature-dependent hydrolysis of alkaline agents, eliminating the tedious and time-consuming step of maintaining a constant pH. Regardless of whether coprecipitation or homogeneous precipitation is used, all precursors are mixed together at the beginning of the reaction, and the supersaturation drops drastically as the reaction proceeds [18], complicating the LDH formation process. The significant overlap between nucleation and growth, possibly involving dissolution/re-precipitation and nanoparticle aggregation, leads to poorly controlled size and size distribution of the products [19,20]. Therefore, the key to improving controllability is to maintain precursor supersaturation at a stable and constant level. Chang et al. established a continuous coprecipitation method using a vortex reactor [21], synthesizing Zn₂Al(OH)₆(CO₃)₀.₅·2H₂O under steady-state conditions; a series of operating parameters were manipulated and investigated to affect the structural and textural properties of the LDHs. However, the absence of post-hydrothermal treatment resulted in lower crystallinity of the as-prepared LDHs compared to those formed via conventional methods. In 2010, Song’s group set up a continuous flow reactor to demonstrate control over nanotube and nanowire growth via a screw-dislocation-driven mechanism [22], which in their subsequent work was proven valid for the growth of 2D materials [23–26], such as Co(OH)₂, Ni(OH)₂, and ZnAl/CoAl LDHs. These configurations, which maintain supersaturation at a constant level, offer a useful alternative to conventional coprecipitation methods, considering their potential to yield LDHs with well-defined morphology.

Among the large family of LDHs, nickel-cobalt (NiCo) LDHs are promising candidates as active materials for high-performance supercapacitors [27], photocatalysts [28], electrochemical catalysts [29,30], and biosensors [31]. In terms of NiCo LDH preparation, widely used methods include electrochemical deposition [32] and coprecipitation/hydrothermal synthesis, with the former intrinsically limited to in-situ growth on conductive substrates. Coprecipitation remains the first choice for large-scale production of NiCo LDH powders, yet the fabrication procedures differ from those of general LDHs. In earlier research, the fabrication of Ni(II)Co(III) LDHs employed a topochemical synthesis method [33,34] involving three steps: (1) preparation of Ni(II)Co(II) mixed hydroxides via coprecipitation (typically using HMTA or urea as precipitating agents) under N₂ gas protection to avoid carbonate contamination; (2) subsequent oxidation of Co(II) to Co(III) using I₂ or Br₂ as topotactic oxidizers; and (3) intercalation of desired anions into LDH interlayers to replace I⁻ or Br⁻ via anion exchange. This tedious process arises from the strong oxidizing nature of hydrated Co(III) ions, which are difficult to stabilize in aqueous environments; thus, the additional conversion of Co(II) in the coprecipitation product to Co(III) is necessary for successful NiCo LDH synthesis. Recently, more feasible procedures have been developed for NiCo LDH synthesis without additional oxidation steps [35,36]. For instance [37], partial oxidation of Co²⁺ to Co³⁺ in mixed NiCo hydroxides can be facilitated by dissolved oxygen in solution when NH₄OH is used as the alkaline agent, via conversion of the [Co(II)(NH₃)₆]²⁺ complex to the more stable [Co(III)(NH₃)₆]³⁺. However, high temperature, pressure, and prolonged heat treatment appear necessary to obtain well-defined NiCo LDHs under these conditions. On the other hand, direct synthesis of NiCo LDHs via conventional hydrothermal methods often yields more amorphous nanoplates when HMTA or urea are used as coprecipitation agents [38].

This work introduces a novel continuous flow reactor (CFR) strategy to synthesize nickel-cobalt layered double hydroxides (NiCo LDHs) under mild conditions with precisely controlled supersaturation, enabling tunable nucleation modes and morphology evolution from 2D nanoplates to 3D nanoflowers. Unlike conventional methods that require harsh oxidation steps or prolonged treatments, our approach achieves high crystallinity and phase purity without additional oxidants or post-synthesis modifications, and systematically correlates supersaturation with mechanistic transitions between homogeneous and heterogeneous nucleation. Moreover, the threshold concentration for homogeneous and heterogeneous growth was delineated by the initial conditions that resulted in nanoplates on substrates and in flowing suspension. Furthermore, we identify key factors—including total concentration, alkalinity, nucleation type, and dissolved oxygen—that strongly influence the phase transformation kinetics from brucite-like intermediates to well-defined NiCo LDHs, offering new insights into the sustainable and controllable synthesis of complex LDH materials for advanced applications.

## 2. Materials and methods

### 2.1. Materials

Nickel nitrate hexahydrate (Ni(NO₃)₂·6H₂O, 99.999%), cobalt nitrate hexahydrate (Co(NO₃)₂·6H₂O, 98%), and hexamethylenetetramine (HMTA, 99.5%) were purchased from Sigma-Aldrich and used without further purification. All other chemical reagents were of analytical purity and used as received. The FTO glass substrates were cleaned by sonication in DI water and ethanol for 20 min.

### 2.2. The CFR setup

The CFR configuration is based on a jacketed chromatography column (25 mm I.D., 300 mm L.) that contains an inner and outer compartment. Using a circulating water bath (Clifton, NE4-14P), a heating solution (1:4 vol.% ethylene glycol: water) is flowed through the outer compartment. The precursor solutions are flowed into the inner compartment via a low-flow peristaltic pump (Heidolph, PD 5101 with Multi-Channel Adaptor).

**Fig 1:**
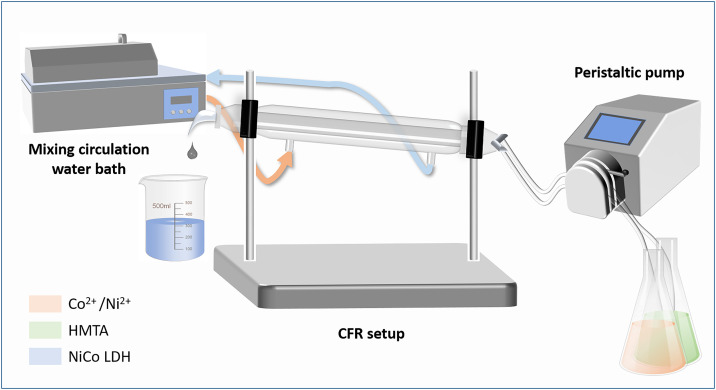
Schematic of the continuous flow reactor system.

During the experiment, the precursor solution is carried by Masterflex® Tygon® tubing (LFL, L/S 16 #06429-16) inward via the entrance and outward through the exit of the column, connected by a Teflon adapter. The reacted precursor solution is finally collected in a waste container. Because the nitrate and HMTA solutions react gradually at room temperature, the two precursor solutions were flowed in separate feeding lines and mixed through a T-junction near the entrance of the column. A schematic diagram of the experimental setup used in this study is presented in **Fig 1**.

### 2.3. Seeds coating process

The seeds are crucial for LDH growth under low supersaturation, especially below the threshold of heterogeneous nucleation. The cleaned substrates were placed in a beaker containing 15 mL of 100 mM Ni(NO₃)₂ and 100 mM HMTA aqueous solution, which was then heated in a 90°C water bath for 1 min. The seed-loaded substrates were then cleaned with DI water and ethanol and dried in an N₂ atmosphere.

### 2.4. Au colloidal suspension fabrication

Au colloidal nanoparticles were prepared by reducing chloroauric acid with borohydride, with the products capped with trisodium citrate [39]. Briefly, 1 mL of aqueous solution A (0.1 M NaBH₄, ice-cold) was added into 25 mL of aqueous solution B (0.4 mM HAuCl₄, 0.12 mM Na₃C₆H₅O₇) under stirring. The mixed solution was then stored in a 2°C refrigerator for 4 h to remove any residual NaBH₄ via reaction with water.

### 2.5. CFR synthesis

The precursor solutions for CFR synthesis were prepared by dissolving Ni(NO₃)₂ + Co(NO₃)₂ and HMTA separately in nanopure water (18.2 MΩ·cm) with identical volumes, while the concentration and metal/alkaline molar ratio were varied to carefully control supersaturation. After the CFR reached the hydrolysis temperature of 95°C, the substrates (with or without seeds) were positioned near the exit port of the column. After reconnecting the waste lines to the CFR, the precursor solutions were pumped to fill the CFR at a fixed flow rate of 4 mL/min. The CFR growth started subsequently and continued for the desired period. Once completed, the precursor solutions and heating flow were stopped, and the samples were removed from the CFR as soon as possible. The reacted substrates were further cleaned with DI water and ethanol and dried under N₂ flow. The free-standing powders in the effluent were also collected by centrifugation at 10,000 rpm for 5 min and rinsed repeatedly with DI water. The details of the CFR synthesis conditions, including the precursor solution, substrates, and duration, are listed in **Table 1**.

**Table 1:** The controlled experimental conditions for a series of samples.

Sample	Precursor Concentration HMTA: Ni(NO_3_)_2_:Co(NO_3_)_2_	Duration	Remark
**1t**	200 μM, 100 μM, 100 μM	8 h	Concentration vs Morphology, Nucleation
**4t**	0.8 mM, 0.4 mM, 0.4 mM	6.5 h	Concentration vs Morphology, Nucleation
**10t**	2 mM, 1 mM, 1 mM	9 h	Concentration vs Morphology, Nucleation
**20t**	4 mM, 2 mM, 2 mM	9 h	Concentration vs Morphology, Nucleation
**50t**	10 mM, 5 mM, 5 mM	4 h	Concentration vs Morphology, Nucleation and Growth; Phase transformation
**50t-1**	5 mM, 5 mM, 5 mM	4 h	Concentration vs Growth; Phase transformation
**50t-2**	50 mM, 5 mM, 5 mM	4 h	Concentration vs Growth; Phase transformation
**50t-Au-1**	10 mM, 5 mM, 5 mM	4 h	Phase transformation; 1% Vol. Au colloidal suspension added in precursor solution
**50t-Au-2**	10 mM, 5 mM, 5 mM	4 h	Phase transformation; 2.5% Vol. Au colloidal suspension added in precursor solution
**100t**	20 mM, 10 mM, 10 mM	4 h	Phase transformation
**200t**	40 mM, 20 mM, 20 mM	4 h	Concentration vs Morphology; Phase transformation

### 2.6. Characterization

The structure and phase of the samples were characterized by X-ray diffraction (Shimadzu powder and thin-film diffractometer with Cu Kα radiation, λ = 1.5406 Å). each sample was measured in triplicate, and the data presented in the graphs are means. Error bars were omitted for the sake of clarity in the figures. The relevant raw data can be found in the dataset file. The sample morphology was examined by scanning electron microscopy (SEM, JEOL 6340F).

## 3. Results and discussion

### 3.1. The effect of precursor concentration on LDH

#### 3.1.1. The effect of concentration on morphology.

**Fig 2:**
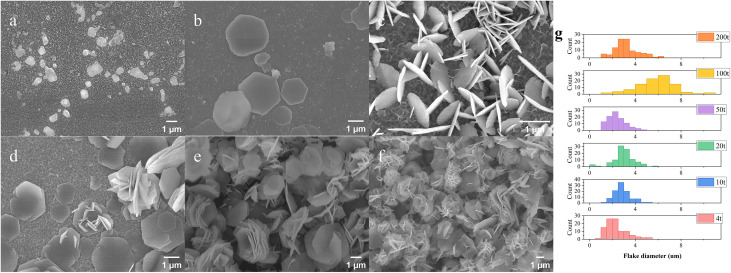
SEM images of nanostructure obtained from the products formed on FTO glass substrates under a series of different super-saturation at a fixed metal cations/HMTA ratio: (a) 1t; (b) 4t; (c) 10t; (d) 20t;(e) 50t; (f) 200t; (g) Histograms of flake diameter for products obtained at different supersaturation levels.

**Fig 2** shows the morphology evolution of the as-prepared products on FTO glass as the overall concentration varies with a fixed molar ratio of metal/alkaline agents. At ultralow supersaturation (total metal ion concentration of 0.2 mM), typical hexagonal LDH nanoplates are absent except for some irregular sheets; given the pre-existing seeds on substrates, this absence might indicate a re-dissolution process competing with simultaneous LDH growth under these conditions. As concentration increases (**Fig 2b**), numerous isolated nanoflakes appear with diameters of a few microns, along with some stacked-disc-like and intersected nanoplates that become more prominent at higher concentrations (**Fig 2d**). The morphological evolution progresses with increasing precursor supersaturation, transforming LDH from well-defined 2D nanoplates to semi-2D rosette structures (**Fig 2e**). At 40 mM total metal ion concentration, abundant 3D nanoflowers composed of assembled LDH sheets dominate the product morphology (**Fig 2f**). The flakes diameters were shown in **Fig 2g** for products obtained at different supersaturation levels.

#### 3.1.2. The effect of concentration on nucleation.

Some previous research has claimed the role of seeds as initial nuclei in the process of LDH synthesis, upon which metal ions/metal hydroxides adsorb, react, and/or incorporate during the evolution and growth of LDH; these seeds could be metal oxide/hydroxide from the precursor [40,41] or those preferentially precipitated due to different *Ksp* in a certain pH range [42–44]. In our case, the seeds on the substrates were introduced to favor LDH growth given a low supersaturation, under which even heterogeneous nucleation might not occur. At an enhanced supersaturation (10t), the presence of LDH nanoplates on the substrate indicates that the supersaturation level has exceeded the critical concentration for heterogeneous nucleation; moreover, plenty of free-standing powder could be collected from the effluent at 50t, as evidence of stable and sufficient homogeneous nucleation in the flowing suspension.

Based on classical nucleation theory [45], the relationship between the Gibbs free energy barriers to form a solid nucleus of critical size for homogeneous and heterogeneous nucleation is shown in Equation 1.


$$\Delta G_{het}=\Delta G_{homo}\times s(\theta )$$ (1)

when s(θ) is a function of the contact angle between the solid nuclei and substrate. Meanwhile, the Gibbs free energy is proportional to 1/Δμ², where Δμ represents the supersaturation, as illustrated in Equation 2.


$$\Delta \mu =\text{kTln}\frac{c}{c_{\text{0}}}$$ (2)

where k is the Boltzmann constant, T is the temperature, and c and c₀ are the actual concentration and equilibrium concentration, respectively. To overcome the Gibbs free energy barrier for the two different types of nucleation, an elevated supersaturation must be provided in homogeneous nucleation. Specifically, the respective concentrations would be as shown in Equation 3.


$$\frac{c_{homo}}{c_{\text{0}}}=({\frac{c_{het}}{c_{\text{0}}})}^{\frac{\text{1}}{\sqrt{s(\theta )}}}$$ (3)

Although not precisely determined, the value of critical concentration could be roughly estimated; as observed in the LDH morphology change during supersaturation increase, the respective concentration ranges are delineated: 1t < c₀ < 4t, 4t < c_het_ < 10t, 20t < c_homo_ < 50t. Therefore, the calculated result of s(θ) ranges from 0 to 0.591, with the corresponding contact angle from 0° to 95°.

#### 3.1.3. The effect of concentration on growth.

The spiral-style growth of LDH has been observed and studied earlier, yet the intrinsic growth mechanism had not been investigated in depth or definitively confirmed until Forticaux’s work [26]; their research clearly revealed that the screw-dislocation driven growth controls the well-defined 2D LDH formation, by maintaining a low and constant supersaturation of precursor through the CFR configuration. Based on the Burton-Cabrera-Frank (BCF) theory for crystal growth [46], the prevalent screw-dislocations on crystal facets could serve as the impetus for the spiral growth of LDH; thereby, unlike the nucleation process, the screw-dislocation driven growth requires no critical supersaturation to overcome the Gibbs free energy barrier. The combination of nucleation and growth mechanisms might explain the morphology evolution corresponding to supersaturation increase.

**Fig 3:**
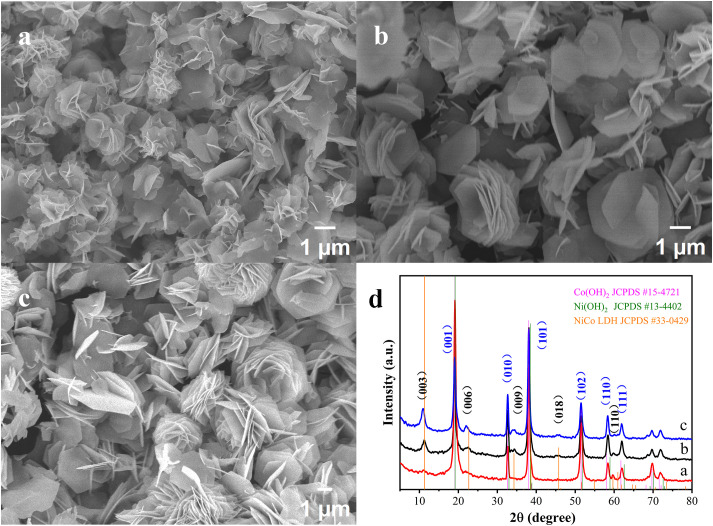
SEM images of nanostructure obtained from the powder samples formed under a series of different metal cations/HMTA ratio at a fixed metal cations concentration at 50t: (a) metal cations/HMTA = 2:1; (b) metal cations/HMTA = 1:1; (c) metal cations/HMTA = 1:5. **(d)** XRD patterns of these three products, respectively.

As precursors enter the reactor, they could participate in both homogeneous and heterogeneous nucleation as well as subsequent LDH growth. The initial concentration that was lower than the equilibrium concentration inhibits LDH yield, possibly due to imbalanced ion adsorption and re-dissolution. When c₀ < c < c_het_, the transient nuclei seldom exceed the critical radius as a prerequisite for stable nucleation, and thus precursors preferentially promote LDH growth around seeds; thereby, well-controlled production of isolated 2D LDH nanoplates can be feasibly realized. As c_het_ < c < c_homo_, higher supersaturation boosts heterogeneous nucleation on substrates and subsequent LDH vertical growth, thus causing stacked/crossed plates, as shown in **Fig 2c**; meanwhile, stacked-disc-like LDHs and intersected LDH sheets might develop from multiple LDH nanoplates centered at the same nuclei, due to relatively low nucleation rates compared to growth rates at medium supersaturation. Furthermore, reaching homogeneous nucleation concentration induces morphological transition from 2D to 3D nanostructures; extreme supersaturation triggers massive secondary nucleation, generating curved nanosheets. Moreover, minimization of high-energy edges drives 3D flower assembly via oriented attachment. Apart from overall precursor increase, similar LDH morphology evolution is shown in **Fig 3a**–**3c** when base/metal ratio increases; at fixed metal ion concentration, elevated HMTA in precursors results not only in higher product yield but also a greater proportion of stacked-disc-like LDH compared to isolated LDH nanoplates.

### 3.2. Phase transformation

#### 3.2.1. Composition analysis.

**Fig 4:**
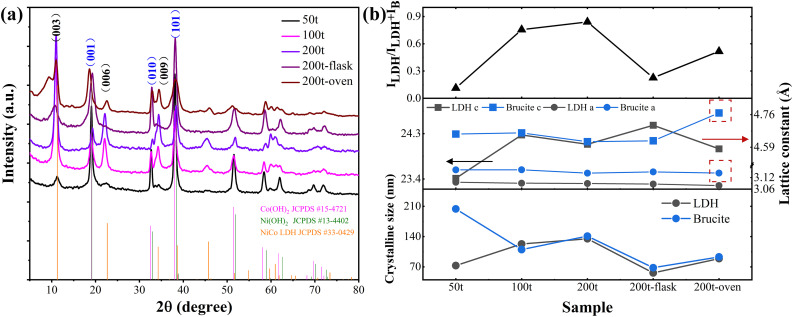
The XRD patterns of the free-standing powders at a series of different supersaturation at a fixed metal cations/HMTA ratio formed in CFR as well as the 200t control sample synthesized by the conventional hydrothermal method in a flask and a Teflon-lined autoclave;. (b) phase ratio, lattice constant and crystalline size of LDH and brucite phase.

The formation of LDH consists of several stages, including the initial formation of Ni and Co hydroxide monomers, the partial conversion of Co²⁺ to Co³⁺ with the aid of dissolved oxygen, and the olation reaction of monomers to form the final LDH [47]. In our case, similar reactions in the CFR should occur due to the identical reactants and reaction temperatures. The phases of the products were examined by XRD to probe the reaction mechanism during LDH nucleation and growth in the CFR. As shown in **Fig 4a**, the powders collected from the flowing suspension exhibit typical XRD diffraction patterns of hydrotalcite structure, characteristic of LDHs; the other major group of peaks corresponds to NiCo hydroxide, a phase regarded as the precursor in conventional topochemical synthesis. The phase and structural parameters of LDH and brucite phase were obtained from the XRD patterns, as shown in **Fig 4b**. When parameter a of the unit cell in brucite phase and LDH is acquired from the respective (110) peaks, the value of c can be calculated from the (001) peak for brucite phase and (003) reflection for LDH. The LDH phase exhibits hexagonal lattice parameters with an *a* value ranging from 3.07 to 3.10 Å, which is smaller than that of the brucite-like phase under the same conditions, primarily due to metal ion oxidation. The *c* value of the brucite-like phase ranges from 4.6 to 4.76 Å. Among the products derived from β-Co(OH)₂-like phases, this value depends mainly on the substitution of Co(II) by Ni(II) within equivalent lattice sites. The variable structural parameters within brucite-like phases might indicate the effect of supersaturation on the composition of NiCo mixed hydroxide during the coprecipitation process. Structural fluctuations also exist among LDH products, particularly in the interlayer distance that expands during the intercalation process.

The coexisting LDH and brucite phases appear to suggest the principal pathway of LDH formation – the phase conversion coupled with oxidation from the initially precipitated brucite phase. Based on this assumption, the degree of phase transition could be evaluated by the intensity of characteristic peaks from different phases; herein, the most intense peaks originating from the LDH and brucite phase, namely the first basal peaks at approximately 11° and 19°, are selected and their respective intensity ratio is also shown in **Fig 4b**.

For comparison, conventional static hydrothermal fabrication of LDH was also conducted to examine the difference between the CFR synthesis and static methods in phase transition. Briefly, 50 mL of precursor with the same concentration as 200t was water-bathed at 90°C for 4 hours in a flask; another 25 mL of solution was heated at 100°C for 16 hours in a Teflon-lined autoclave. Estimated from the XRD peaks, the sample prepared in the flask exhibits the lowest ratio of LDH to brucite phase, while the heat treatment in the autoclave promotes phase conversion remarkably due to enhanced temperature, pressure and duration. However, the products of the CFR system complete the phase transition and achieve the highest degree of total LDH synthesis in comparison with the two static methods, with the largest LDH grain size among three samples. The sharp and symmetric diffraction peaks of CFR-synthesized LDH reveal an additional advantage of the CFR system over static methods in achieving better LDH crystallinity, as opposed to the broad XRD peaks in the static LDH sample.

#### 3.2.2. The influencing factors of phase evolution.

Supersaturation is one of the factors that determine the LDH phase evolution during CFR synthesis. As illustrated in **Fig 4b**, increased supersaturation could effectively enhance the LDH/brucite ratio in the final product; for example, prominent LDH peaks coupled with minor residual brucite phase are detected at 200t, indicating complete phase transition. This phenomenon might imply the role of supersaturation as the driving force in the phase transition process, and/or the effect of NH₄OH derived from HMTA hydrolysis to stabilize Co³⁺ and thus beneficially improve the LDH conversion rate. The latter reason also explains the mild increase in the LDH/brucite ratio with higher HMTA concentration at fixed metal ion concentration, as shown in **Table 2**.

**Table 2:** Lattice parameters, grain size and the phase conversion ratio calculated from the XRD patterns for powder samples formed at varying conditions.

Sample	Phase ratio	c(LDH) (Å)	a(LDH) (Å)	LDH grain size (nm)	Brucite grain size (nm)
50t	0.11	23.506	3.096	76	205
50t-1	0	–	–		157
50t-2	0.19	24.137	3.077	90	155
50t-Au-1	0.53	23.745	3.066	96	160
50t-Au-2	0.76	23.549	3.069	93	138
100t	0.75	24.273	3.091	123	110
200t	0.84	24.088	3.089	135	141
200t-flask	0.22	24.466	3.086	56	68
200t-oven	0.52	23.998	3.079	89	93

**Fig 5:**
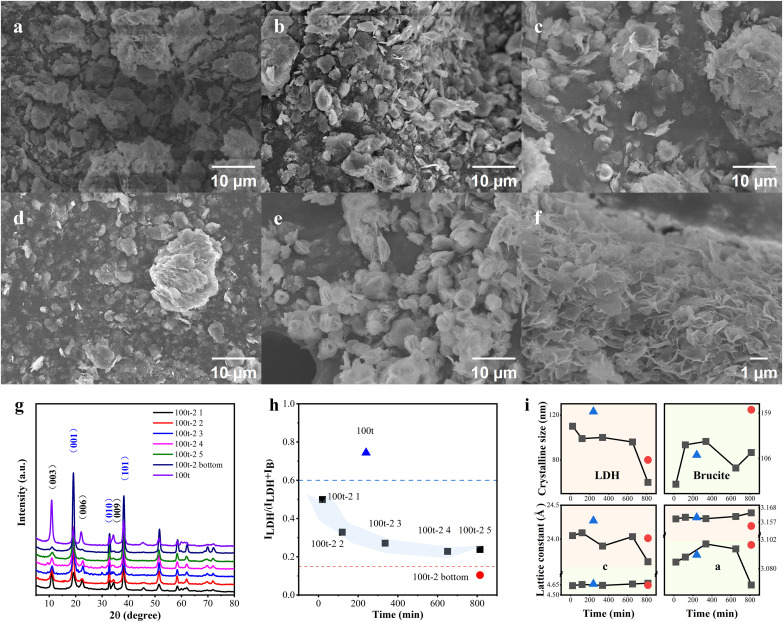
SEM images of the products collected from the effluent continuously at a fixed supersaturation of 100t with the flowing rate of 2 mL/min: (a-d) 100t-2 1 to 100t-2 5, (e) 100t-2 bottom and (f) 100t. **(g)**The XRD patterns of the as-mentioned samples with flow rate at 2 mL/min, in comparison with the 100t sample with flow rate at 4 mL/min; **(h)** The phase conversion rate of sample vs. the sample collecting time from the start of the experiment till 800 mins at flow rate of 2 mL/min, the red dot refers to the sample collected from the CFR bottom after the whole experiment ends; the blue dot refers to the phase conversion of 100t sample at 4 mL/min collected at 250 mins; **(i)** the crystalline sizes and lattice constants of LDH and brucite-like phase, respectively.

Another key factor appears to be the flow rate of CFR, which was further explored through a long-term experiment with a slower flow rate. Briefly, the precursor with 100t concentration was fed into CFR at a flow rate of 2 mL/min (instead of 4 mL/min as above), while the duration was extended from 4 to 14 hours. Samples were continuously collected from the flowing suspension at specific intervals and named sequentially as 100t-2 1 to 100t-2 5. As shown in **Fig 5a**–**5f**, the flakes in 100t-2 samples appear larger than those in 100t, which might be explained by the longer growth duration of 100t-2 samples with slower flow rate. XRD analysis was then conducted to examine the phase conversion stability, as shown in **Fig 5g**–**5i**. Unlike the counterpart product collected at fast flow rate, the brucite-related peaks appear more pronounced while weaker LDH peak intensities are observed at slow flow rate, suggesting depressed phase conversion under this condition. Notably, powders formed after 800 minutes exhibit negligible LDH characteristic peaks. The sample (100t-2 bottom) collected from the sediment in the CFR tube shows a low percentage of LDH phase. The overall phase conversion ratios are reduced due to the slower flow rate causing higher HMTA hydrolysis (increased OH⁻) that leads to precipitation of higher brucite phase content per unit volume, with incomplete conversion to LDH under depleted oxygen content at sluggish flow. Moreover, the grain size of the LDH and the interlayer spacing decreased as the reaction time lengthened, resulting in smaller values compared to those of the 100t sample.

**Fig 6:**
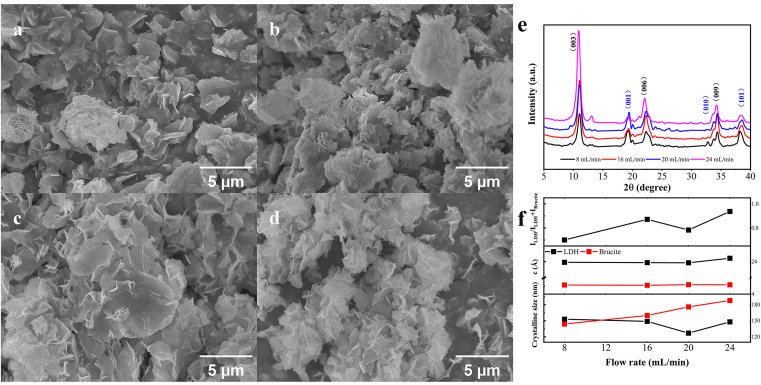
SEM images of nanostructure obtained from the free-standing powders formed at a fixed precursor concentration (200t) under different flow rate: (a) 8 mL/min; (b) 16 mL/min; (c) 20 mL/min; (d) 24 mL/min. **(e)** XRD patterns of these four products, respectively; **(f)** phase ratio, lattice constant c and crystalline size of LDH and brucite phase.

The amount of dissolved oxygen becomes insufficient to induce oxidative-intercalated phase transformation, accompanied by increased brucite phase formation. On the other hand, the milder flow might result in oxygen-deficient zones at the CFR bottom due to weaker solution convection and gas diffusion, providing a possible explanation for the ultralow LDH/brucite phase ratio in the powders; the massive production of brucite phase at the bottom in turn influences the later-formed samples in the flow, lowering the phase transformation efficiency. The relationship between flow rate and phase conversion was further clarified by plotting the XRD patterns of products under various flow rate, as shown in **Fig 6e**–**6f**. As the flow rate increases, the extent of phase transformation is gradually enhanced as well as the interlayer spacing, in good agreement with the aforementioned explanation. As shown in **Fig 6a**–**6d**, the powders exhibit similar shapes and sizes except for those produced at a flow rate of 24 mL/min, which are highly aggregated, probably due to flow fluctuations at high speed [48]. An interesting phenomenon is the increasing grain size of the brucite-like phase as its phase ratio decreases. This may suggest that smaller brucite grains are more advantageous for LDH phase evolution under oxygen-rich conditions, while larger grains remain relatively unchanged.

**Fig 7:**
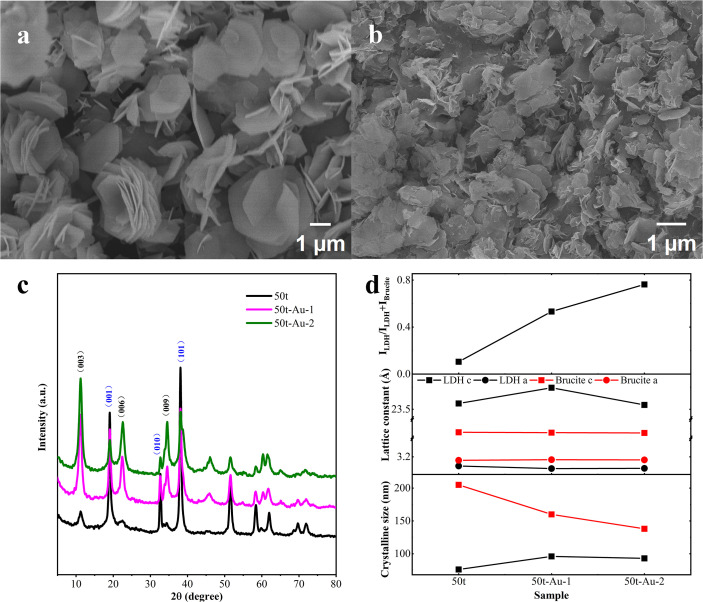
SEM images of nanostructure obtained from the powder products formed at a fixed supersaturation at 50t without/ with Au nanoparticles added in the precursor solution: (a) 50t and (b) 50t-Au-2. **(c)** The XRD patterns of 50t, 50t-Au-1 and 50t-Au-2, respectively; **(d)** phase ratio, lattice constant c and crystalline size of LDH and brucite phase.

The investigation above on the LDH/brucite phase is mainly based on the free-standing powder, which primarily originates from homogeneous nucleation; in order to explore the impact of nucleation type on phase transition, Au nanoparticles were added to the precursor to provide heterogeneous nuclei for LDH growth. The XRD patterns (**Fig 7c**) reveal that the Au nanoparticles in the flowing suspension appear to strongly support the transformation from brucite phase to LDH phase, or to some extent suppress the yield of brucite phase under conditions of heterogeneous nucleation (**Fig 7d**). Meanwhile, the morphology of products originating from the precursor containing Au seeds (**Fig 7b**) tends to yield more isolated nanoplates with smaller sizes, possibly due to more nucleation sites in the flowing suspension and fewer shared core centers for screw-dislocation driven growth, further reducing the size and dimensions of nanoplates.

**Fig 8:**
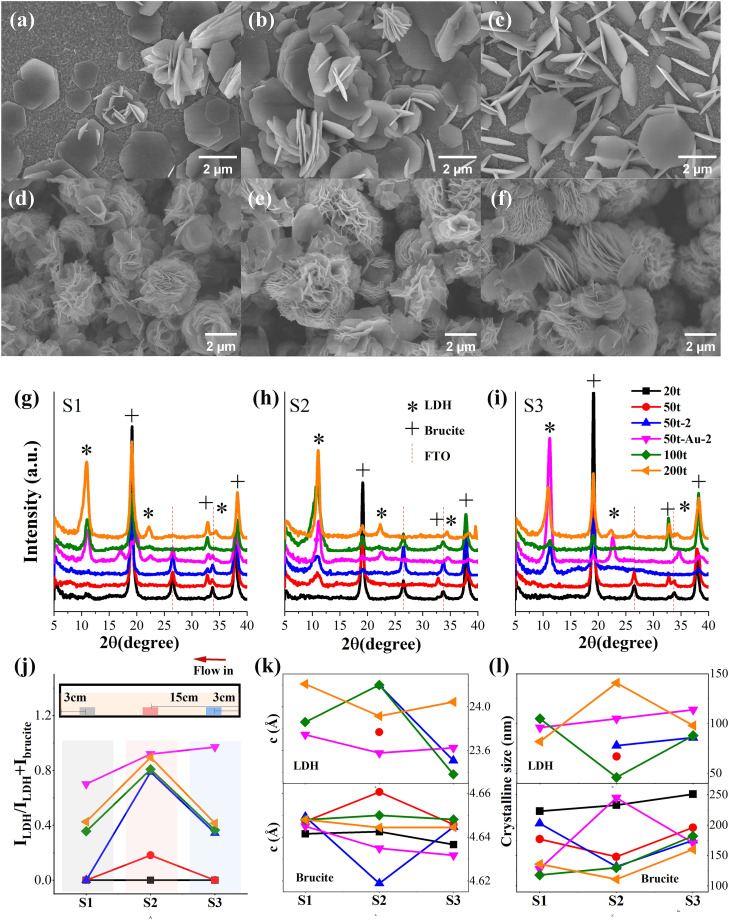
SEM images of nanostructure in (a) 20t-S1, (b) 20t-S2, (c) 20t-S3, and (d) 200t-S1, (e) 200t-S2, (f) 200t-S3; XRD patterns of nanostructure obtained from the products grown on FTO glasses under varying conditions at three positions: (g) S1, 3 cm to the exit of CFR, (h) S2, in the center of column and (i) S3, 3 cm to the entrance of CFR; (j) LDH ratio, (i) lattice constant c and (l) grain size of sample at different positions.

The phase transition process was further analyzed in terms of sample spatial distribution. Along the column axis, the positions of the FTO substrates were adjusted to near the exit, the entrance, and the middle, which were denoted as S1, S3, and S2, respectively. The as-formed films on FTO glass were examined by XRD, as shown in **Fig 8**.

The three samples at a concentration of 20t provide noticeable characteristic signal from the brucite phase with negligible LDH peaks, again emphasizing the essential role of supersaturation in phase conversion as stated above; the morphology, grain size and lattice constants of brucite phase were similar among the S1-S3 samples, as shown in Fig 8a–8c. Like the free-standing powders, the elevated supersaturation also fosters the LDH formation on the FTO substrates, yet the conversion ratio is significantly uneven among the samples in different positions. The specimens in the center of reactor yield a higher LDH/brucite rate as compared to the ones near two ends of the column, except the Au seed samples. When the concentration increased to 50t, the LDH phase first appeared at the central position. As the concentration further rose to 100t, the LDH content at positions S1–S3 significantly increased, while at 200t, the pro-portion of LDH showed little change. Interestingly, at both 100t and 200t, the d-spacing and grain size exhibited opposite trends across different locations: at 100t, the central sample had the smallest grain size yet the largest interlayer spacing, whereas at 200t, the central sample showed the largest grain size but the smallest interlayer spacing. Moreover, although differences in LDH ratio and grain size were observed, the 200t samples showed little morphological variation across different locations, as shown in **Fig 8d**–**8f**.

Furthermore, increasing the proportion of HMTA also significantly enhances the LDH content, like the case observed in powder samples. However, the alkaline solution primarily influences the d-spacing, with less impact on the grain size of both LDH and brucite. This suggests that during heterogeneous nucleation on the FTO surface, it mainly pro-motes the conversion of brucite to LDH, thereby increasing the proportion of LDH. The introduction of gold as a seed plays a more significant role in enhancing the LDH ratio than increasing concentration or adjusting the HMTA pro-portion. In contrast to the effect of alkaline solution, the addition of gold reduces the d-spacing while increasing the grain size. Moreover, it notably improves the consistency of the LDH phase across different locations.

## 4. Conclusions

In summary, the continuous and controllable synthesis of NiCo nanoplates was successfully realized by using conventional reagent metal ion salts and HMTA as feeding precursors in the CFR system. In the CFR system, supersaturation could be feasibly maintained at a constant level to better control the morphology and size of LDH; more importantly, the relatively steady-state condition enables investigation of the relationship between supersaturation and nucleation method, LDH growth, and subsequent morphology changes. The threshold concentration for homogeneous and heterogeneous nucleation was first delineated, and coupled with crystal growth, the morphology evolution was discussed in correspondence to supersaturation increase. Moreover, the phase transition from brucite phase to final LDH structure was studied based on conversion rates under various supersaturation conditions and positions, for both free-standing powders and products on substrates. The overall supersaturation, metal/alkaline ratio, heterogeneous nucleation, and dissolved oxygen are confirmed as key factors driving the transformation of NiCo mixed hydroxides to NiCo LDH. This study provides insights into direct and scalable fabrication of NiCo LDH materials, particularly highlighting the impact of well-controlled supersaturation and heterogeneous nucleation.

## Supporting information

S1 Data The dataset enclosed within all pictorial representations. (XLSX)

## Abbreviations

LDH: layered double hydroxide
CFR: continuous flow reactor
